# Age and Sex-Based Differences in Therapy Preferences Among Patients with Cancer: Evidence from a Novel Therapy Preference Scale

**DOI:** 10.21203/rs.3.rs-5743881/v1

**Published:** 2025-01-08

**Authors:** Prajwal Dhakal, Christopher S. Wichman, Bunny J Pozehl, Radowan Elnair, Amulya Yellala, Kalika Mahato, Vijaya Raj Bhatt

**Affiliations:** The University of Iowa; University of Nebraska Medical Center; University of Nebraska Medical Center; Roger Williams Medical Center; University of Nebraska Medical Center; University of Nebraska Medical Center; University of Nebraska Medical Center

**Keywords:** Cancer, Patient, Therapy, Preference, Quality of life, Life expectancy

## Abstract

**Background:**

Patients’ preferences are crucial to formulating personalized treatment plans. We developed a self-reported questionnaire, Therapy Preference Scale (TPS), to examine treatment preferences of patients with cancer.

**Methods:**

TPS has 30 questions-19 on patients’ preferences on safety, quality of life, and treatment effectiveness, 8 questions on importance of various treatment characteristics, and 3 on patients’ preferred intent of therapy, expenses, and life expectancy gain. We recruited 300 adults>18 years with a cancer diagnosis and categorized them into 4 age-by-sex groups (younger men<60 years, older men≥60 years, younger women<60 years, older women≥60 years). Kruskal-Wallis non-parametric ANOVA method was used to compare responses among 4 groups.

**Results:**

Older women, compared to other groups, were more likely to value maintenance of cognitive function and ability to perform daily activities. Older women were more likely to risk having a shorter life expectancy rather than facing the risk of long-term cognitive impairments. Younger men and women were more likely to accept more effective treatment, even if it caused significant pain. A higher percentage of younger men, compared to other groups, valued maintaining sex life. Older men and women were more likely to prefer oral pills over intravenous therapy.

**Conclusion:**

Patient preferences may vary based on age and sex with older women prioritizing cognitive and physical function independence more frequently, whereas younger men and women willing to pursue more effective treatment options even with a greater risk of significant pain. TPS can elicit detailed preferences of an individual patient to facilitate shared decision-making with their oncologists.

## Introduction

Cancer treatment decisions are complex and involve weighing the benefits and risks of different treatment options and each patient’s unique preferences and values. Treatment options depend on various patient, disease, and treatment characteristics, and personalized treatment plans require shared decision-making between the patient and physician. Patients’ preferences can vary depending on their treatment goals, concerns about potential side effects, the impact of treatment on their daily lives, and their desired quality of life. Additionally, factors such as the individual’s age, functional and cognitive abilities, family support, financial status, and clinician recommendations can impact these preferences [1]. Understanding patients’ preferences is critical to ensure patients receive treatment that aligns with their goals and expectations.

To better understand how patients consider and express their preferences for efficacy, safety, and treatment burden, we developed a self-reported questionnaire, Therapy Preference Scale (TPS) [2–5]. In our prior publications, we have described the development of TPS in detail and preliminary data on 100 patients with cancer.[2–5] In this study, we used TPS to examine the treatment preferences of a diverse group of patients with cancer across different age and sex groups. Our aim was to investigate how patient preferences for cancer treatments vary by age and sex and to identify areas of similarity and difference in treatment preferences across these groups. We hypothesized that there would be significant differences in treatment preferences depending on age and sex and understanding these differences could help formulate patient-centered cancer treatment strategies.

## Patients and Methods

### Therapy Preference Scale

TPS is a self-reported questionnaire comprising 30 questions designed to assess patients’ preferences for different aspects of cancer treatment (**Supplement File**) [2–5]. Briefly, out of 30 questions, 19 measure patients’ importance ratings for safety, quality of life, and treatment effectiveness on a 1–10 scale. Eight questions gauge the significance of various treatment characteristics (on a 4-item Likert scale with choices of ‘strongly disagree,’ ‘disagree,’ ‘agree,’ and ‘strongly agree.’). Three additional questions identify patients’ preferred intent of therapy, maximum acceptable out-of-pocket expenses, and minimum life expectancy gain required to undergo treatment.

### Patient enrollment and data collection

We enrolled adult patients more than 18 years of age with a cancer diagnosis who presented to the University of Nebraska Medical Center from July 2019 to May 2021. Patients were enrolled during outpatient clinic visits, or hospital stays at Fred and Pamela Buffett Cancer Center and were asked to fill TPS based on their current treatment preferences. Demographic, cancer, and therapy-related data were collected from the patient and electronic health records for analysis. The institutional review board at the University of Nebraska Medical Center approved the study.

### Statistical analysis

Descriptive statistics were used for patient characteristics. We divided patients into 4 age-by-sex groups: male (M) <60 years, M ≥60 years, female (F) <60 years, and F ≥60 years. The intent of treatment (curative vs. palliative) was compared across the groups using Pearson’s Chi-squared test). The responses to questions on “Safety and Quality of life,” “Effectiveness of cancer treatment,” and “Treatment characteristics” were measured on an ordinal 1–10 scale. We compared the responses to these questions across the age-by-sex groups using the Kruskal-Wallis (KW) non-parametric ANOVA method. The questions on “Treatment preferences” were measured on a 4-level ordinal scale on strongly disagree, disagree, agree to strongly agree. Due to the ordinal nature of the data, the responses were recoded as 1 through 4, with Strongly Disagree = 1, Disagree = 2, Agree = 3, and Strongly Agree =4, and then compared across the age-by-sex groups using the KW non-parametric ANOVA method. Questions on maximum acceptable out-of-pocket expenses and minimum life expectancy gain required to undergo treatment were measured in dollars and years, respectively. We also compared responses between M <60 and M ≥60 and F <60 and F ≥60. Responses were categorized into 2 groups: “disagree” (combining “strongly disagree” and “disagree”) and “agree” (combining “strongly agree” and “agree”).

## Results

### Patient characteristics

Among the 300 patients, median age was 61 years (range 19–89 years), 51% were female, and 91% were white (Table 1). Sixty-two percent of patients were diagnosed with solid tumors, whereas 38% had hematological malignancies. Thirteen percent of patients had a history of more than 1 type of cancer. Thirty-nine percent of total patients were in remission, 54% were not in remission, and 7% had a new diagnosis of cancer and had not started any treatment yet. Median age for M <60 was 49 years (range 21–59 years), and 68 years (range 60–89 years) for M ≥60. Median age for F <60 was 49.5 years (range 19–59 years) and was 67 years (range 60–83 years) for F ≥60. At the time of survey, 61% of patients were receiving curative intent treatment compared to 39% of patients on palliative treatment as determined by the treating physician. When compared using Pearson’s Chi-squared test, there was no detectable association in the intent of treatment types between the 4 age-by-sex groups (χ^2^ = 4.03; p = 0.2).

### Safety and quality of life

**Figure 1:**
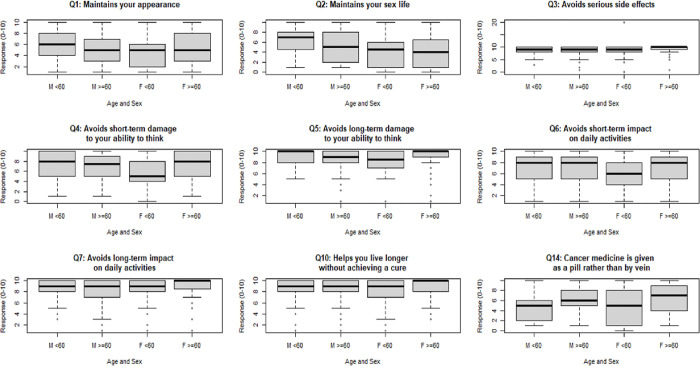
Questions with differences in preferences among 4 age-by-sex groups on an ordinal 1–10 scale

Table 2 details the responses to the questions capturing patients’ rating of the importance of safety, efficacy, and other characteristics of systemic cancer treatment on a scale of 1–10. Eight out of 19 questions indicated a significant difference in opinion among the 4 age-by-sex groups (Figure 1). These questions queried about maintaining sex life (KW statistic= 22.2, p=0.0001), avoiding serious side effects (KW= 15.5, p=0.01), avoiding short-term damage to ability to think (KW= 11.6, p=0.008), avoiding long term damage to ability to think (KW=15.5, p=0.001), avoiding short term impact on daily activities (KW=12.3, p=006), and avoiding long term impact on daily activities (KW=0.01, p=0.007), living longer without cure (KW=11.1, p=0.01), and oral rather than intravenous therapy (KW=12.3, p=0.006). Box plots for questions whose K-W test p-value was ≤ 0.10 are presented in Figure 1.

A higher percentage of patients in M <60 group valued maintaining sex life compared to other groups (Table 3). Compared to F<60 and M<60, a higher proportion of F ≥60 and a lower proportion of M ≥60 would avoid long-term impact on daily activities. A greater number of F ≥60 and M ≥60 preferred oral pills to intravenous therapy. A higher proportion of F ≥60 placed more importance on long-term ability to think, remember things, and make decisions. Avoiding short-term impact on daily activity and avoiding long-term ability to think were important to a lower percentage of F <60 compared to other groups.

### Effectiveness of cancer treatment

Among the most preferred treatment goals (cure, increased life expectancy, or symptom relief), 49.0% of total patients valued cure the most, 30.3% valued increased life expectancy, and 6.3% valued symptom relief; 14.3% of patients did not answer the question (**Table 4**). There were no significant differences in the preferred outcomes among the 4 age-by-sex groups (χ^2^ = 9.0; p = 0.4).

### Treatment preferences

**Figure 2:**
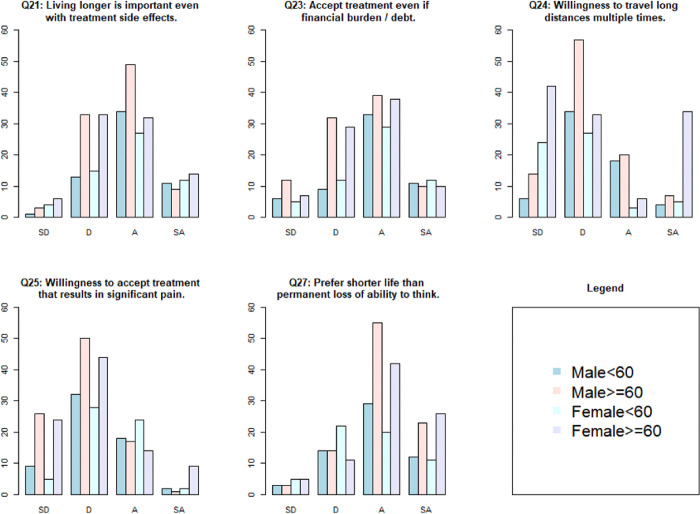
Questions with differences in preferences among four age-by-sex groups on a 4-level ordinal scale

**Table 5** details the responses to the 8 questions that measured importance of different aspects of treatment with a 4-level ordinal scale (strongly disagree, disagree, agree, strongly agree). Four of the 8 questions showed differing opinions among the 4 age-by-sex groups (Figure 2). Figure 2 depicts all questions with p-value ≤ 0.1. These questions queried about accepting a very effective treatment which may result in a financial burden including debt (KW=9.4, p=0.02), traveling long distance multiple times during treatment (KW=12.6, p=0.005), undergoing more effective treatment that may cause significant pain (KW=20.4, p=0.0001), and living a shorter life than permanently lose ability to think, remember things, and make decisions (KW=12, p=0.007).

Among the 4 groups, financial debt was less important for a higher percentage of M <60, while shorter travel distance was important for M ≥60 (Table 3). A greater number of M≥ 60 valued quality of life over living longer. A higher proportion of M <60 and F <60 valued more effective treatment even if the treatment could cause significant pain (e.g., mouth sores or stomach cramps). More F ≥60 were willing to accept shorter life expectancy rather than risk permanent loss of ability to think, remember things, and make decisions.

### Out-of-pocket expenses and life expectancy

An out-of-pocket expense of more than $5000 was unacceptable to 34% of all patients, whereas 25% would pay $20000 or more for an effective treatment. Forty-nine percent responded that at least 12 months increase in life expectancy would make it worthwhile to consider a harsh cancer treatment. There was no statistical association between age-by-sex categories and maximum acceptable out-of-pocket or minimum expected life-expectancy gain to undergo harsh cancer treatment.

## Discussion

In this study, we utilized a novel self-reported questionnaire, demonstrating that cure was the top priority for patients of all ages and sex with 4/5th of patients prioritizing cure or increased life expectancy. Although patients would be willing to accept short-term side effects to improve life expectancy, more than 2/3rd of patients preferred maintenance of cognition, functional ability, and quality of life over increased life expectancy. About half of the patients would want a minimum of 12 months of gain in life expectancy to accept a harsh treatment. In addition, reducing treatment burden on caregivers was important. Several of these preferences were similar regardless of the age and sex of the patients.

Our study also identified differences in preferences, mainly in relation to side effects and treatment characteristics based on age and sex. Although cure was the top priority for all age and sex groups, two-thirds of younger patients prioritized cure compared to only half of older patients. Younger patients were more likely to accept treatment that helped them live longer despite the potential negative impact on quality of life, cognitive and functional ability, and higher financial burden. While living longer was important, older patients were more likely to prefer a better quality of life, fewer side effects, preservation of cognitive ability, and oral instead of intravenous treatment.

In our study, we directly compared patients in four subgroups based on age and sex, which has not been explored in detail in patients with cancer. One systemic review assessed studies that used different methods of preference elicitation to summarize patients’ priorities, with focus on 70 years or older patients with cancer. The results identified quality of life as the topmost priority than overall survival in more than 3/4th of total patients. One study in a non-cancer patient population showed that females were less likely to prefer life-sustaining therapies (e.g., cardiopulmonary resuscitation, surgery, and artificial feeding) and more likely to choose a dignified death than males [6]. Female patients with cancer are reported to prefer palliative care focused on comfort rather than aggressive cancer treatment compared to male patients [7, 8]. In breast and ovarian cancer studies, patients would trade off a decrease in survival for less severe treatment-related toxicity [9–11]. In one study on breast cancer, older women, compared to younger counterparts, preferred quality of life and independence over chemotherapy with side effects and minimal survival benefit [1]. Literature shows that most older patients prioritize health outcomes such as maintaining cognitive ability, functional status, and freedom from pain and other symptoms over increased survival [9, 12, 13]. Also, older patients often prefer less treatment burden, such as shorter hospital stays, shorter travel distance and proximity of healthcare centers, minimally invasive interventions, and oral rather than intravenous therapy, consistent with our results [12].

Our study shows that our multidimensional questionnaire, TPS, can be an important tool to assess and analyze the treatment preferences of individual patients of different age and sex. During cancer diagnosis and treatment, patients are faced with many decisions. These decisions may be ‘big,’ such as selecting a treatment strategy at diagnosis or cancer progression or making advance directives, or relatively ‘small,’ such as choosing between oral and intravenous treatment options. Patients’ approaches and responses to these decisions may vary over time, even if they repeatedly face the same question. TPS can provide valuable insight into patients’ preferences and goals of care at different time points, helping to personalize treatment plans. In addition, by comparing TPS responses at different time points, clinicians can anticipate and address specific areas of utmost importance to each patient. TPS can also facilitate deeper engagement between patients and clinicians, which is important as patients who are more engaged in their treatment decisions often have better treatment outcomes and higher levels of satisfaction with their care.

Our single-center study has both strengths and limitations. Most participants had already received treatment, and the responses may differ during treatment and based on anticipated outcomes. However, our patient population was all-inclusive, with a similar proportion of male and female participants of different age groups with varying types of cancer. We used TPS with specific questions tailored to efficacy, side effects, treatment characteristics, out-of-pocket expenses, and increased life expectancy. Additionally, the enrollment of 300 patients at a single center within a year and the short completion time of 10 minutes for TPS indicates that the use of TPS is feasible and well-received by patients.

## Conclusion

Our study demonstrated age- and sex-based differences in preferences to avoid side effects, preserve cognitive and functional ability, and maintain sex life, and differences in preferences towards receiving an oral treatment. The study findings underscore the importance of understanding both shared and distinct treatment preferences based on the age and sex of the patients. TPS can elicit detailed preferences of an individual patient, which is necessary to facilitate shared decision-making between patients and their oncologists. Future research should investigate how patients and providers perceive TPS in regular clinical practice, compare TPS to other tools, and evaluate its impact on shared decision-making through clinical trials. Additionally, it will be important to assess the patient preferences for specific cancer types and examine how these preferences may evolve over the treatment course.

## Figures and Tables

**Table 1:** Patient responses to questions on ordinal scale of 1–10

Questions	Male <60 (M, Q1-Q3)	Male ≥60 (M, Q1-Q3)	Female <60 (M, Q1-Q3)	Female ≥60 (M, Q1-Q3)	p-value
Maintains your appearance	6 (4–8)	5 (3–7)	5 (2–6)	5 (3–8)	0.07
Maintains your sex life	7 (4.5–8)	5 (2–8)	4.5 (1–6)	4 (1–6.25)	0.0001
Avoids serious side effects	9 (8–10)	9 (8–10)	9 (8–10)	10 (9–10)	0.001
Avoids short-term damage to your ability to think, remember things and make decisions	8 (5–10)	7.5 (5–9)	5 (4–8)	8 (5–10)	0.008
Avoids long-term damage to your ability to think, remember things and make decisions	10 (8–10)	9 (8–10)	8.5 (7–10)	10 (9–10)	0.001
Avoids short-term damage to your ability to do daily activities such as grooming, eating, or self-care.	8 (5–19)	8 (5–9)	6 (4–8)	8 (5–9)	0.006
Avoids long-term damage to your ability to do daily activities such as grooming, eating, or self-care	9 (8–10)	9 (7.25–10)	9 (8–10)	10 (8.5–10)	0.007
Helps you live longer without necessarily achieving a cure?	9 (8–10)	9 (8–10)	9 (7.25–10)	10 (8–10)	0.01
Offers the chance of a cure	10 (9–10)	10 (8–10)	10 (9–10)	10 (9–10)	0.3
Relieves your symptoms such as fatigue, pain, or shortness of breath without necessarily increasing your life expectancy or achieving a cure	7 (5–9)	8 (6–10)	8(5–9)	8 (5–10)	0.3
Cancer medicine is given as a pill rather than by vein	5 (2–6)	6 (5–8)	5 (1.25–8)	7 (4–9)	0.006
Treatment is available in a clinic close to your home	8 (7–10)	8 (7–10)	8 (7–10)	9 (8–10)	0.2
Treatment is associated with a short or no hospital stay	8 (5–9)	8 (7–9)	7.5 (5–9)	9 (5–10)	0.1
Treatment limits the number of invasive procedures necessary for making treatment decisions	7 (5–9.75)	7 (5–8)	6 (4–9)	8 (5–10)	0.2
Treatment does not significantly disrupt your lifestyle	7 (5–9)	7 (5–8)	6 (4.25–8)	8 (5–8.5)	0.1
Cost you pay for treatment, such as out-ofpocket expenses is affordable	8 (6.25–10)	9 (6.25–10)	8 (6.25–10)	8.5 (6.75–10)	0.7
Treatment does not result in a significant burden to your family or friends (for example, due to care necessary at home or for getting you to doctor visits	8 (7–10)	8 (6–10)	8 (6–9)	9 (7–10)	0.1

M= median, Q1– 1^st^ quartile, Q3– 3^rd^ quartile

**Table 2:** Patient responses to questions on treatment characteristics

	Male<60 (agree, %)*	Male≥60 (agree, %)*	Female<60 (agree, %)*	Female>≥60 (agree, %)*
Living longer is important to me even if it will result in side effects such as life-threatening infection	78	58	66	50
Living longer is important to me even if treatment will result in poor quality	42	22	37	31
I would accept a treatment that is very effective but results in a financial burden including debt	75	49	62	62
I would travel long distance (for example, 2 hours or more) multiple times during treatment to receive care from cancer experts	90	79	90	89
I would undergo a more effective treatment even if the treatment causes significant pain	83	69	88	64
I would undergo treatment that maintains or improves my quality of life but does not help me live longer	67	78	73	72
I would rather live a shorter life than permanently lose my ability to think, remember things and make decisions	72	83	62	80
I would rather live a shorter life than permanently lose my ability to do daily activities such as grooming, eating, or self-care	74	81	67	77

* The results indicate the proportion of patients who agreed with the statements. ‘Strongly agree’ and agree’ responses were categorized into ‘agree’ and ‘strongly disagree’ and ‘disagree’ responses were categorized into ‘disagree’ for the analysis.

**Table 3:** Patient responses to questions on treatment characteristics on 4-level ordinal scale (Strongly disagreed, Disagree=2, Agree=3, Strongly agree=4)

Questions	Male<60 (M, Q1-Q3)	Male≥60 (M, Q1-Q3)	Female<60 (M, Q1-Q3)	Female≥60 (M, Q1-Q3)	p-value
Living longer is important to me even if treatment will result in side effects such as life-threatening infection.	3(3–3)	3(2–3)	3 (2–3)	3 (2–3)	0.08
Living longer is important to me even if treatment will result in poor quality of life.	2 (2–3)	2 (2–2.75)	2 (2–3)	2 (2–3)	0.1
I would accept a treatment that is very effective but results in a financial burden including debt.	3 (2.5–3)	3 (2–3)	3 (2–3)	3 (2–3)	0.02
I would travel long distance (for example, 2 hours or more) multiple times during treatment to receive care from cancer experts.	3 (3–4)	3 (3–3)	3 (3–4)	3(3–4)	0.005
I would undergo a more effective treatment even if the treatment causes significant pain (for example, mouth sores, or stomach cramps).	3(3–4)	3 (2–3)	3 (3–4)	3 (2–3)	0.0001
I would undergo treatment that maintains or improves my quality of life but does not help me live longer.	3 (2–3)	3 (3–3)	3 (2–3)	3 (2–3)	0.4
I would rather live a shorter life than permanently lose my ability to think, remember things and make decisions.	3 (2–3)	3 (3–3)	3 (2–3)	3 (3–4)	0.007
I would rather live a shorter life than permanently lose my ability to do daily activities such as grooming, eating, or self-care.	3 (2–3.5)	3 (3–3)	3 (2–3)	3 (3–4)	0.1

1 = Strongly Disagree; 2 = Disagree; 3 = Agree; 4 = Strongly Agree, M= median, Q1– 1^st^ quartile, Q3– 3^rd^ quartile

## Data Availability

The data from this study will not be shared openly to protect study participants privacy and may be available from the corresponding author upon reasonable request.
